# Improvement of *Stevia rebaudiana* Bertoni *In Vitro* Propagation and Steviol Glycoside Content Using Aminoacid Silver Nanofibers

**DOI:** 10.3390/plants11192468

**Published:** 2022-09-21

**Authors:** Mariana Sichanova, Maria Geneva, Maria Petrova, Kameliya Miladinova-Georgieva, Elisaveta Kirova, Trendafil Nedev, Daniela Tsekova, Iwan Iwanov, Konstantin Dochev, Viktoria Ivanova, Antoaneta Trendafilova

**Affiliations:** 1 Institute of Plant Physiology and Genetics, Bulgarian Academy of Sciences, Acad. G. Bonchev Street, Bldg. 21, 1113 Sofia, Bulgaria; 2 Department of Organic Chemistry, University of Chemical Technology and Metallurgy, 8 St. Kl. Ohridski Blvd, 1756 Sofia, Bulgaria; 3 Institute of Organic Chemistry with Centre of Phytochemistry, Bulgarian Academy of Sciences, Acad. G. Bonchev Street, Bldg. 9, 1113 Sofia, Bulgaria

**Keywords:** antioxidant activity, *in vitro* propagation, nanofibers, carrier of Ag particles, *Stevia rebaudiana* Bert.

## Abstract

The food industry is interested in replacing artificial sweeteners with natural sugars that possess zero calories and carbohydrates and do not cause spikes in blood sugar levels. The steviosides leaves, synthesized at *Stevia rebaudiana* Bertoni, are 300 times sweeter than common table sugar. *Stevia* propagation is limited due to the poor viability of the seeds, the long time and low germination rate, and the poor rooting ability of vegetative cuttings. Because of this, an alternative biotechnological method for its reproduction is being studied, such as multiple shoot production through direct organogenesis using nanofibers, formed from a derivative of amino acid valine as a carrier of the biologically active agent silver atoms/particles (NF-1%Ag and NF-2%Ag). The stevia explants were cultured on a medium containing NF-1%Ag and NF-2%Ag at concentrations of 1, 10, 50, and 100 mg L^−1^. The NF-1%Ag and NF-2%Ag treatment caused hormetic effects on stevia plantlets. At low concentrations of from 1 to 50 mg L^−1^ of nanofibers, the stimulation of plant growth was observed, with the maximum effect being observed at 50 mg L^−1^ nanofibers. However, at the higher dose of 100 mg L^−1^, inhibition of the values of parameters characterizing plant growth was recorded. The presence of nanofibers in the medium stimulates stevia root formatting.

## 1. Introduction

Since ancient times, people have mainly used plants to treat various diseases. Pharmacy has created modern contemporary medicines by studying the biologically active properties of secondary metabolites in plants. The production of a large percentage of current therapeutic agents is based on the use of natural products derived from medicinal and aromatic plants. Therefore, it is necessary to cultivate medicinal and aromatic plants in conditions under which certain biologically active secondary metabolites necessary for the pharmaceutical, cosmetic, and food industries will be synthesized.

Stevia (*Stevia rebaudiana* Bertoni) [1] is a valuable medicinal plant of the Asteraceae family with wide application in the pharmaceutical and food industry. The food industry is increasingly interested in replacing artificial sweeteners with other natural sugars in order to offer the consumer a wider range of choices and to satisfy the requirements of a segment of the population that does not want to or cannot eat sucrose. The stevia leaves have been used as a low-calorie sweetener for centuries, and are currently consumed worldwide. The sweetness of this plant is due to the accumulation of bioactive compounds, especially diterpenoid steviol glycosides, which are up to 30% of the leaf’s dry weight [2]. The steviosides are 300 times sweeter than common table sugar, with the additional advantages of having zero calories, zero carbohydrates, not causing spikes in blood sugar levels, having a non-fermenting ability, and maintaining thermal stability at 100 °C, as well as a long shelf life [3].

Conventional methods of stevia propagation are limited due to the poor viability of seeds, prolonged germination, low germination rate, and poor rooting ability of vegetative cuttings [4,5]. All this is a prerequisite for reducing the ability of plants to survive in natural conditions when propagated by seeds. To respond to the growing demands for this powerful medicinal plant with a declining population and help its conservation, suitable alternative biotechnological approaches (less time-consuming, less somaclonal variation, and high success rate) for its reproduction are being studied, such as multiple shoot production through direct organogenesis [6,7]. It has been found that various factors influence the success of *in vitro* cultivation—the initial explants, genotype, nutrient medium composition, and the combination and concentration of plant growth regulators.

Most protocols concerning *S. rebaudiana* multiplication used different explants—leaf, nodal, internodal segments, and shoot tips for *in vitro* culture—and involved supplementation with cytokinins (for shoots initiating) and auxins (for rooting) [8,9]. Cytokinins (BAP, kinetin, TDZ) alone or in combination with auxins (NAA, IAA, IBA) promote the formation of shoots, as the maximum multiplication efficiency is observed on nutrient media containing BAP [10,11].

Often during the micropropagation of plants, the development of microorganisms appears as well as browning and necrosis of plant tissues due to phenolic exudation in the medium [12,13]. Silver nanoparticles are a suitable additive used for removing microbial contaminants in plant tissue culture [14]. Several studies have represented data that AgNO_3_ possessed a beneficial effect on various plant species’ regeneration [15,16,17]. It is well known that AgNO_3_ acts as an ethylene inhibitor. The ethylene and the polyamines use the same precursor for their biosynthesis, S-adenosyl methionine (SAM) [18]. Consequently, inhibiting the ethylene action by AgNO_3_ will enhance polyamine biosynthesis. There are hypotheses that AgNO_3_ inhibits ethylene action because the silver ions replace copper ions located in the hydrophobic pocket of the ethylene receptors leading to the reducing the receptor capacity to bind ethylene [19]. This would lead to the accumulation of ethylene in the tissues till ethylene production reaches its maximum level, which inhibits its own biosynthesis, and the use of the precursor SAM will turn to polyamine biosynthesis [20]. It has been shown that in somatic embryogenesis in carrots, the inhibiting of ethylene production by AgNO_3_ leads to an increase in the levels of endogenous polyamines in carrot embryogenic cultures [21]. Polyamines have been shown to enhance plant growth and development as well as basic biological processes [22].

The literature survey revealed that there are limited data on changes in antioxidant activity and the quantity and quality of biologically active secondary metabolites in *in vitro* cultivated *S. rebaudiana* caused by growth regulators and silver nanoparticles added to the nutrient media, but most reports concern their use on undifferentiated callus tissues. There are studies in the literature about the effect of salicylic acid, silver nanoparticles, ZnO, and CuO on callus growth and biologically active secondary metabolites production [23,24]. Another report on the effect of the silver nanoparticles on *in vitro* multiplication of stevia has recently been published, with results obtained for their transport and accumulation in plant tissues [25].

Currently, preparing functional objects with at least one dimension in the range of 1 to 100 nm^2^ is the focus of very active research, as these materials have potential applications in many fields. Self-assembly is a convenient way to synthesize structure size from nanometers to micrometers, where molecules are highly organized. Among the challenging shapes of nanosized objects are self-assembled fibers and fibrils.

Here we suggest an application of nanofibers from a low molecular compound derivative of the amino acid valine as promising carriers of colloidal silver particles for plant cultivation. The amino acids are another important factor influencing plant growth because most of the nitrogen is bound up to them. During *in vitro* propagation, the addition of amino acids to the MS nutrient medium provides a primary fast source of nitrogen to plants as compared to inorganic nitrogen. An increase in formation and elongation of the cell wall and cell division was observed due to the addition of amino acids. To control oxidative browning during *Rosa centifolia* micropropagation, ascorbic acid, citric acid, and activated charcoal may be added to the MS medium supplemented with BAP and NAA, while different concentrations of glutamine, asparagine proline are used to control the withering of shoots [26]. There are several studies in the literature about the effect of amino acids on plant propagation *in vitro*. There is no information about their effect if they are bound in molecules that self-aggregate in nanosized fibers, which are carriers of biologically active agents.

**Figure 1:**
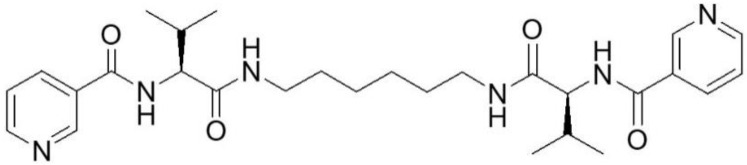
Structure of the compound M6.

For the purpose described, we use a compound we have designated as M6 (compound **1**) (Figure 1), which synthesis and capability to produce fibrilar networks in organic solution and also in the absence of solvent are firstly reported by Tsekova et al. [27]. This compound contains a bolaamphiphile structure and includes two fragments of valine and nicotinic acid linked together and doubled through diamino hexane spacer.

The pyridine moiety in this type of molecule is believed to provide a basis for incorporating metal particles onto the fibers [28,29]. Crystalline nano fibrillar material obtained from compound **1** was soaked in a solution with colloidal silver nanoparticles and left for solvent evaporation, expecting that the silver nanoparticles would adhere to and among the fibers. As compound **1** possesses extremely low solubility in water, it can be regarded as a heterogeneous additive that slowly releases adhered in colloidal silver. The *in vitro* studies of this compound in white mice revealed low oral and intraperitoneal toxicity (over 2000 mg kg^−1^ b.w.) and a lack of prolonged toxicity [30].

This study envisaged the preparation of nanofibers from low molecular weight peptidomimetics and their use as a delivery system for silver nanoparticles when added at various concentrations to the MS medium. This is a completely new area, and there is no data in the literature regarding the influence of silver attached to nanofibers on the morphological characteristics and antioxidant activity of micropropagated plants. Many questions about the *in vitro* cultivation of the studied plant for obtaining biomass with higher antioxidant activity are still unresolved and await answers.

Based on the above, the present study aims to describe an efficient protocol for direct shoot regeneration from nodal explants of *Stevia rebaudiana* Bert by using a new type of nanofibers formed by newly synthesized low molecular weight peptidomimetics carriers of the biologically active agent silver atoms/particles. This *in vitro* propagation method could be used for commercial scale propagation and conservation of uniform plantlets of Stevia in a relatively short period.

## 2. Results

### 2.1. Organic Compounds Synthesis and Analysis

Two organic compounds have been isolated and purified—the intermediate one and the targeted final one—(M6) (Figure 1). They have been analyzed through ^1^H and ^13^C NMR spectra and have the following signals:

Compound Boc-Val6: C_26_H_50_N_4_O_6,_ white solid (89% yield). ^1^H NMR (400 MHz, DMSO) δ *=* 7.80 (t, J *=* 5.7 Hz, 1H), 6.55 (d, J *=* 9.0 Hz, 1H), 3.74—3.65 (m, 1H), 3.04 (ddd, J *=* 24.4, 13.2, 6.7 Hz, 2H), 1.87 (dd, J *=* 13.5, 6.5 Hz, 1H), 1.37 (s, 11H), 1.23 (s, 2H), 0.81 (dd, J *=* 6.5, 4.8 Hz, 6H) ppm. ^13^C NMR (101 MHz, DMSO) δ 171.65, 155.85, 78.44, 60.29, 38.71, 30.78, 29.41, 28.61, 26.39, 19.64 ppm.

Compound M6: C_28_H_40_N_6_O_4,_ white solid (65% yield). M.p. 275 °C. ^1^H NMR (300 MHz, D_6_-DMSO) δ *=* 0.88 (6H, d, J *=* 5 Hz), 0.91 (6H, d, J *=* 5 Hz), 1.25 (4H, m), 1.37 (4H, m), 2.08 (2H, m), 3.01 (4H, m), 4.24 (2H, t, J *=* 8 Hz), 7.46 (2H, dd, J *=* 5 Hz J2 *=* 1 Hz), 8.00 (2H, t, J *=* 6 Hz), 8.20 (2H, dt, J1 *=* 8 Hz, J2 *=* 2 Hz), 8.48 (2H, d, J *=* 9 Hz), 8.68 (2H, dd, J1 *=* 5Hz, J2 *=* 2 Hz), 9.00 (2H, dd, J1 *=* 2 Hz, J2 *=* 1 Hz) ppm. ^13^C NMR (500 MHz, D_6_-DMSO) δ *=* 19.53, 19.97, 26.68, 29.64, 30.71, 39.04, 59.88, 123.96, 130.53, 135.95, 149.34, 152.48, 165.74, 171.26 ppm).

**Figure 2:**
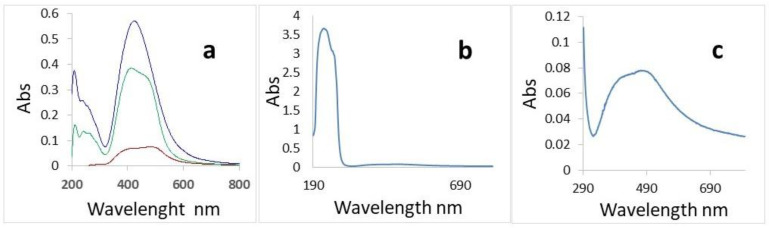
UV study of the used materials. (**a**) Samples from the stock solution from commercially obtained AgNPs.: 0.50 mg mL^−1^, 0.33 mg/mL, and 0.20 mg mL^−1^ in distilled water; (**b**) Absorption of the material prepared as 2% AgNPs on organic compound M6 in ethanol; (**c**) Focus on the absorbance of AgNPs in (**b**).

UV study of the diluted aqua solution of AgNPs showed bands confirming its nature and related to the concentrations of the nanoparticles (Figure 2a). On the other hand, the UV spectrum of the used filamentous compound charged with NF-2% Ag in ethanol shows signals for both parts—organic and silver (Figure 2b,c).

**Figure 3:**
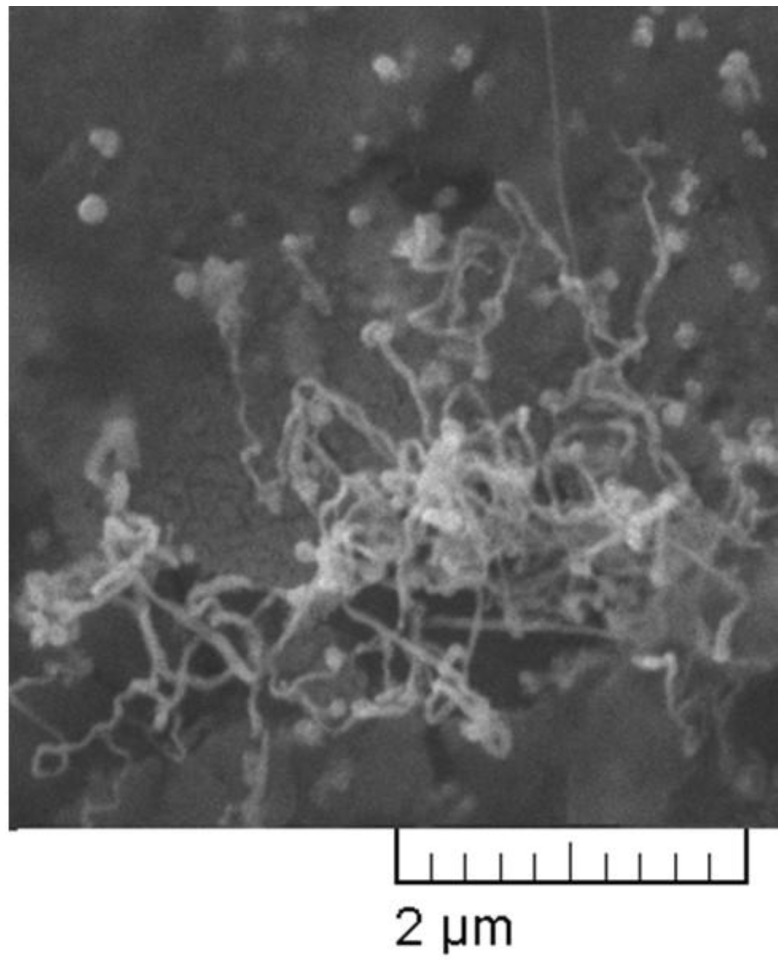
SEM image of fibers with nanosized diameter and silver particles (2%).

Analyzing data presented in Figure 2b,c, we can conclude that the sizes of the nanoparticles are in a broad range reaching 100 nm. SEM image confirms this suggestion (Figure 3). The picture shows that the diameter of most of the colloidal particles is around 100 nm.

**Figure 4:**
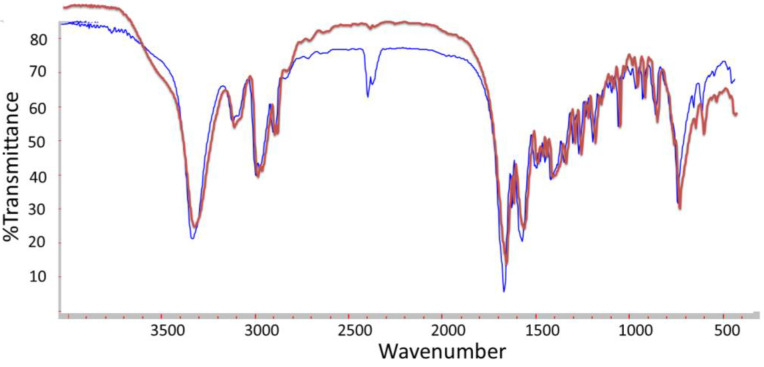
IR spectra of the pure M6 (red) and M6 carrying 2% Ag NPs (blue).

Very few and weak differences can be distinguished in IR spectra of both—complexes and pure organic compounds (Figure 4). It can be addressed to the very low amount of Ag in the sample as far 2% in 5 mg (0.1 mg) is quite low for the apparatus sensitivity. In Figure 4, the increased intensity in some bands could be assigned to Ag signals and interactions between Ag nanoparticles and fibers.

### 2.2. Micro Propagation of S. rebaudiana

The obtained results showed that adding the MS nutrient medium at different concentrations (1, 10, 50, 100 mg L^−1^) of nanofibers, formed by low molecular weight peptidomimetics enriched with 1% and 2% colloidal silver, has a significant effect on the parameters characterizing growth and antioxidant activity as well as stevioside and rebaudioside A content of micropropagated stevia plantlets.

The effect of peptidomimetics nanofibers enriched with 1% and 2% colloidal silver (NF-1%Ag, NF-2%Ag) in MS nutrient media at different concentrations on the growth of *in vitro* micropropageted *S. rebaudiana* plants were tested (Table 1, Figure S1). The organogenesis efficiency was 100% on all examined nutrient media. Among the tested concentrations of nanofibers containing 1% Ag, the best results regarding fresh biomass, length, and shoot numbers per explant were obtained on MS medium containing 50 mg L^−1^ NF-1%Ag. The highest percentage of rooted shoots (42.92%) was achieved on the same medium (Table 1). The explants grown on a control MS medium free of BAP and other additives showed the lowest biomass yield and shoot length, and failed to produce new auxiliary shoots per explant within the tested culture period. Supplementation with NFs enriched with 2% colloidal Ag at increasing concentrations from 1 to 50 mg L significantly augmented the shoot length, fresh biomass yield, and shoot number per explant compared to both control treatments. Maximum shoot production (FW 0.464 g) was achieved on an MS medium containing 50 mg L^−1^ NF-2% Ag. Using nutrient media fortified with 10 or 50 mg L^−1^ NF-2% Ag resulted in the highest average number of shoots per explant (3.25–3.35), which exceeded even the control with cytokinin BAP where 1.7 shoots per explant were observed. Comparing the growth characteristics when adding NF-1%Ag and NF-2%Ag to the MS nutrient medium, it was observed that NF-2%Ag had a greater effect on the growth of stevia plantlets. The level of the growth parameters increased until 50 mg L^−1^ NF-1%Ag and NF-2%Ag, then at 100 mg L^−1^ NF-1%Ag and NF-2%Ag decreased. Therefore, when applied at lower doses, peptidomimetics NFs stimulate the plants’ growth characteristics, though by increasing the concentration, growth is inhibited, which indicates the presence of a hormetic effect. At both control micro plantlets (C and C+BAP), no roots were recorded, but when the nanomimetics NF-Ag were added to the MS we noted root initiation. This demonstrates that nanofibers promote rooting initiation.

### 2.3. Antioxidant Power

**Figure 5:**
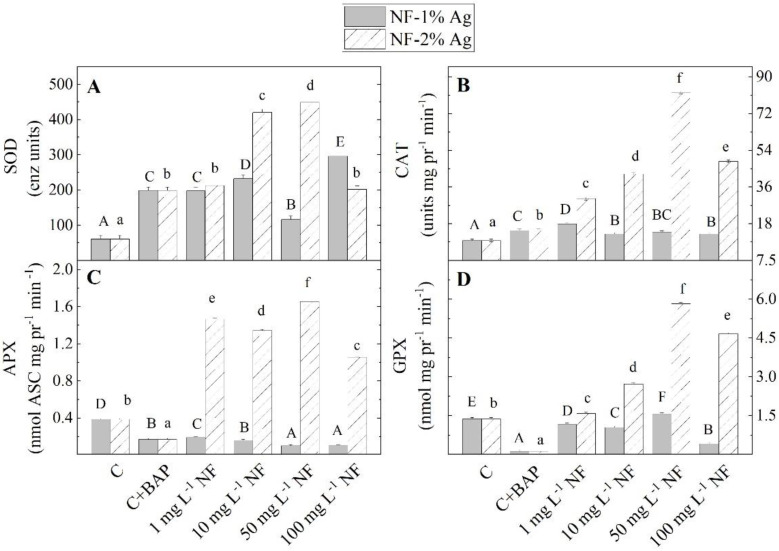
The activity of antioxidant enzymes superoxide dismutase (SOD) (**A**), catalase (CAT) (**B**), guaiacol peroxidase (GPX) (**C**), and ascorbate peroxidase (APX) (**D**) in Stevia rebaudiana plantlets *in vitro* propagated on MS medium, with BAP, and on MS medium supplemented with various concentrations (1, 10, 50, 100 mg L^−1^) of aminoacid nanofibers enriched with 1% and 2% colloidal Ag (NF-1%Ag, NF-2%Ag). Values are means ± SE, n = 20; different letters indicate significant differences assessed by the Fisher LSD test (*p* ≤ 0.05) after performing ANOVA one-way analysis. We used the letter ‘a’ or “A” for the lowest data value and ascending to the next for higher data values. The statistical analysis of NF-1%Ag (uppercase) and NF-2%Ag (lowercase) was performed separately.

The level of antioxidant enzyme activity (SOD and CAT) was increased when plants were *in vitro* propagated on MS nutrient media supplemented with BAP compared to control plants cultured on an MS-free medium (Figure 5). Enzymes (SOD, CAT, APX, and GPX) were with lower activity after NF-1% Ag was added to the MS medium, in comparison with NF-2%Ag. The increased SOD and CAT activity was recorded in plantlets grown with NF-1%Ag, than control untreated plants. In contrast, the activity of peroxidases APX and GPX was decreased. The presence of NF-2%Ag in the MS medium caused the amplification of antioxidant enzyme activities compared to control plants. These activities were increased on 1, 10, and 50 mg L^−1^ NF-2%Ag, while adding 100 mgL^−1^ decreased enzyme activities. In terms of enzymatic antioxidant activity, higher activities of SOD, CAT, APX, and GPX were recorded in *S. rebaudiana* plants grown on media supplemented with NF-2%Ag compared to plants grown on MS with NF-1%Ag.

**Figure 6:**
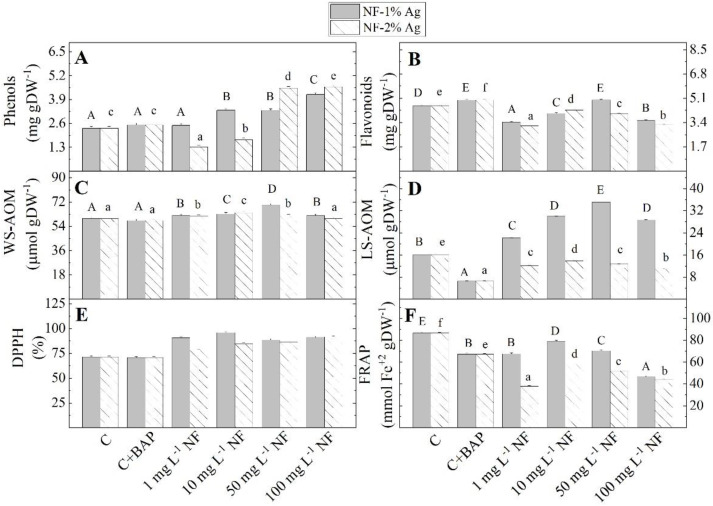
The content of metabolites with antioxidant power (total phenolic compounds (**A**) and flavonoids (**B**), WS-AOM (**C**) and LS-AOM (**D**)) and antioxidant potential (DPPH (**E**), FRAP (**F**)) in *S. rebaudiana* plantlets *in vitro* propagated on MS medium, on MS medium with BAP, and on MS medium supplemented with various concentrations (1, 10, 50, 100 mg L^−1^) of aminoacid nanofibers enriched with 1% and 2% colloidal Ag (NF-1%Ag, NF-2%Ag). Values are means ± SE, n = 20; different letters indicate significant differences assessed by the Fisher LSD test (*p* ≤ 0.05) after performing ANOVA one-way analysis. We used the letter ‘a’ or “A” for the lowest data value and ascending to the next letters for higher-data values. The statistical analysis of NF-1%Ag (uppercase) and NF-2%Ag (lowercase) was performed separately.

The plantlets grown on MS media supplemented with 1 to 100 mg L^−1^ NF-1%Ag showed higher total antioxidant activity measured by radical scavenging capacity (DPPH method) and higher content of total phenols, WS-AOM, and LS-AOM, than controls plants (Figure 6). In contrast, the level of ferric reducing antioxidant power (FRAP method) and the total flavonoid content decreased. The highest antioxidant activity levels measured by DPPH and FRAP methods were recorded in *in vitro* plantlets cultured on MS medium supplemented with 10 mg L^−1^ NF-1%Ag-95.944% and 79.04 mmol Fe^2+^ g DW^−1^, respectively.

The addition of 50 mg L^−1^ NF-1%Ag to the MS medium caused the highest total flavonoid content, water- and lipid-soluble metabolites with antioxidant capacity in stevia plantlets. The highest content of total phenols (4.170 mg g DW^−1^) was obtained in plantlets grown on a nutrient medium supplemented with 100 mg L^−1^ of NF-1%Ag. The supplementation of the MS medium with 1, 10, 50, or 100 mg L^−1^ NF-2%Ag also led to a decrease in the FRAP, total flavonoids, and LS-AOM compared to control plants. The highest level of total flavonoid content, WS-AOM, LS-AOM content, and the highest FRAP activity were assayed when 10 mg L^−1^ NF-2%Ag was added to the MS medium, compared with other investigated concentrations. However, with increasing the concentration of NF-2%Ag from 50 to 100 mg L^−1^ the level of these parameters decreased. Only DPPH scavenging activity and TPC were highest at 100 mg L^−1^ NF-2%Ag.

**Figure 7:**
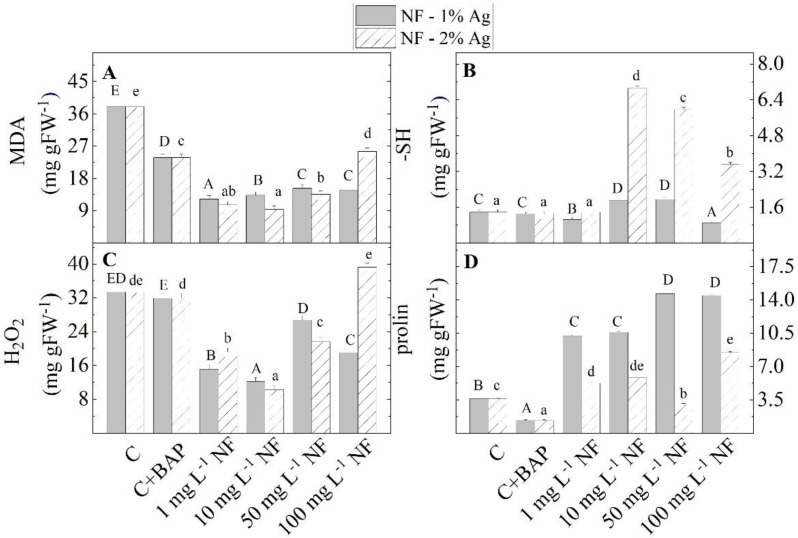
The levels of stress markers (MDA (**A**), -SH groups (**B**), H_2_O_2_ (**C**), and proline (**D**)) in the *S. rebaudiana* plantlets *in vitro* propagated on MS medium, on MS medium with BAP, and on MS medium supplemented with various concentrations (1, 10, 50, 100 mg L^−1^) of aminoacid nanofibers enriched with 1% and 2% colloidal Ag (NF-1%Ag, NF-2%Ag). Values are means ± SE, n = 20; different letters indicate significant differences assessed by the Fisher LSD test (*p* ≤ 0.05) after performing ANOVA one-way analysis. We used the letter ‘a’ or “A” for the lowest data value and ascended to the next letters for higher data value. The statistical analysis of NF-1%Ag (uppercase) and NF-2%Ag (lowercase) was performed separately.

It was found that stress markers such as proline, malondialdehyde (MDA), and H_2_O_2_ decreased when plants were micropropagated in an MS medium supplemented with BAP (Figure 7). In general, the addition of NF-1%Ag to the MS medium at all studied concentrations caused an additional decrease in MDA and H_2_O_2_ content compared with the control untreated plants. The addition of all tested concentrations of NF-2% Ag showed different effects on oxidative stress than the addition of NF-1%Ag. At 100 mg L^−1^ NF-2%Ag, the content of stress markers drastically increased, and the content of SH- groups decreased compared with other tested concentrations. An increase in proline content in the presence of NF-Ag, with the only exception at 50 mg L^−1^ NF-2%Ag, compared to control was observed, and this increase was more significant under NF-1%Ag. The maximum values of this stress marker were enriched at higher NF-1%Ag concentrations (50 and 100 mg L^−1^). In contrast, the amounts of SH-groups are much higher in plants treated with NF-2%Ag than NF-1%Ag treated plants.

### 2.4. Stevioside, Rebaudioside A, and Total Sugar Content

A decrease in the content of stevioside and rebaudioside A was recorded in *S. rebaudiana* plantlets grown with BAP, compared with non-treated control plants, while the content of total soluble sugar did not change (Table 2). The addition of 1–50 mg L^−1^ NF-1% Ag did not significantly change stevioside content in comparison to control untreated plants, but increased in comparison to BAP treated plants. The highest stevioside content was recorded at 100 mg L^−1^ NF-1%Ag. Unlike stevioside, the amount of rebaudioside A decreases compared to untreated controls but remains higher than in plants grown on MS containing BAP (C + BAP) except for 10 mg L^−1^ NF-1%Ag.

All investigated concentrations of NF-2%Ag lowered stevioside and rebaudioside A content compared to control and BAP-treated plants. However, the level of the soluble sugar was recorded to change differently.

## 3. Discussion

The present study showed that *S. rebaudiana* plants react to nanofibers enriched with silver in different ways depending on the percentage of silver in this complex. Treatment with peptidomimetic nanofibers enriched with 1 and 2% colloidal Ag showed that low amounts from 1 to 50 mg L^−1^ of NF-1%Ag and NF-2%Ag stimulated growth parameters and antioxidant activity. In a study conducted by Vishwakarma et al. [31], silver uptake and accumulation in roots were greater when Brassica was treated with AgNO_3_ compared to silver nanoparticles treatment. Therefore, AgNO_3_ caused more significant inhibition of APX and CAT activity, root hair distortion, and changes in root morphology which retarded plant growth to a greater extent than silver nanoparticle treatment.

Numerous reports of the positive effect of AgNO_3_ on plant growth *in vitro* are available in the literature [32,33,34]. Kumar et al. [35] made a detailed overview of possible mechanisms by which silver nitrate affects plant morphogenesis. The most common explanation is that AgNO_3_ acts as an ethylene inhibitor. This action is due to the silver ion, which can replace the copper ion from the ethylene-binding side of the ethylene receptor [36]. There is much literature data concerning the different influences of silver NPs on the growth characteristics of various plant species. Almutairi and Alharbi [37] reported a positive effect of AgNPs on seed germination of watermelon, corn, and zucchini. However, another study has noticed that AgNP application did not affect growth parameters in wheat seedlings [38]. It can be assumed that when silver is introduced into the nutrient medium as salt (AgNO_3_), silver ions are released very quickly into the medium, which slows down plant growth. When silver is added and bound to peptidomimetic nanofibers, it is likely to be more slowly released into the medium, and this does not affect plant growth significantly. Still, it is necessary to conduct more research.

Increasing the amount of nanofiber to 100 mgL^−1^ led to growth inhibition of stevia plantlets. That dose-response phenomenon where low doses induce stimulation and high doses induce inhibition is called hormesis [39]. For example, through the synthesis of auxins and activation of antioxidant defense, some herbicides applied at low concentrations regulate plant growth and alleviate plant stress [40], whereas in high doses, it inhibits plant growth [41].

On the other side, the concentrations from 1 to 50 mg L^−1^, the content of sweet diterpene glycosides stevioside in stevia plantlets treated with NF-1%Ag was almost the same as in control untreated plants. Even when NF-2%Ag was added to the MS nutrient medium, the content of stevioside and rebaudioside A was lower than in the control plants. There was a significant decrement of stevioside and rebaudioside A content in stevia plantlets when adding BAP or NF-Ag2% at MS nutrient media compared to MS medium. Röck-Okuyucu et al. [42] established a significant decrement in stevioside content after adding BAP and other cytokinins (Kn or TDZ) to the culture medium. In their study, rebaudioside A was detected only on the PGR-free control medium. These, along with the higher shoot formation under BAP treatment, led the authors to assume that the secondary metabolism was suppressed at the expense of the primary one. In our study, the same correlation between glycoside content and growth parameters was observed. A close, negative correlation between the content of stevioside and rebaudioside A has been reported [43]. In fact, the last step in the steviol glycosides biosynthetic pathway is the conversion of stevioside into rebaudioside A [44]. AgNPs have a positive effect on the expression of key genes involved in the stevioside and rebaudioside A biosynthesis pathways [45]. Increasing the concentration of silver nanoparticles (0, 10.0, 20.0, 40.0 mM) in the spraying solution has led to an increase in the number of glycosides in the leaves of greenhouse-grown stevia.

The addition of BAP to the nutrient medium did not show a significant effect on total soluble sugar content. However, supplementation with NF-1% and even, to a greater extent, NF-2%, results in a remarkable increase in this parameter. Furthermore, the soluble sugar content in the presence of NF-2% was changed in a concentration-dependent manner with a maximum value of 94.9 mg g FW^−1^ under 100 mg L^−1^ NF (with 15.25 mg g FW^−1^ for the control variant). An increase in soluble sugar content was reported by Nokandeh et al. [46] after treatment of greenhouse-grown stevia plants with different concentrations of AgNPs. Total soluble sugars are well known to play an important role in maintaining cell homeostasis [47]. A strong correlation between soluble sugar content and stress tolerance has been reported [48].

When plants are exposed to harmful environmental conditions, such as radiation, high or low temperature, or some harmful substances, plants generate reactive oxygen species. The content of this oxygen species in plants is regulated by fine-tuned enzyme and non-enzyme antioxidant defense systems [49]. During *in vitro* propagation in tubes, plants are in harmful environmental conditions, such as high humidity, high plant growth regulator (PGR) content, and low light [50]. Under stress conditions in plant tissues are multiplied the generation of reactive oxygen species, such as superoxide, hydrogen peroxide, and hydroxyl radicals, may cause cell damage. To mitigate and repair the damage, plants possess enzyme and non-enzyme mechanisms that detoxify reactive oxygen species. Therefore, experiments were conducted for the comparison of the enzyme antioxidant capacity and stress markers of *in vitro* propagated stevia plants in Murashige and Skoog (MS) media supplied with 1, 10, 50 100 mg L^−1^ nanofiber formed from peptidomimetics as a carrier of Ag ions (NF-1%Ag, NF-2%Ag). The study demonstrates the benefits of using nanofibers formed by peptidomimetics and enriched with silver to accelerate the growth and antioxidant potential of Stevia in *in vitro* plants. From the decreasing of the content of the lipid peroxidation marker MDA and stress marker hydrogen peroxide in stevia plantlets cultivated in MS media with a low concentration of NF-1%Ag and NF-2%Ag, and the respective increase of the enzymes with antioxidant potential (SOD, CAT, APX), it could be concluded that NF-1%Ag and NF-2%Ag reduced oxidative stress in *S. rebaudiana* plantlets during *in vitro* propagation. The results of our study demonstrate that the defense mechanisms of stevia plants were activated by adding AgNF at different concentrations. Therefore, it is possible that the growth-stimulating effect could be due to the action of the amino acids used to form the nanofibers or the silver. A similar trend has been observed by Fazal et al. [51], which reported enhanced total protein content and the activity of superoxide dismutase and peroxidase in callus cultures of Prunella vulgaris L when MS has been supplied with AgAu (1:3) or Au nanoparticles.

The differences in the activity of antioxidant enzymes when comparing the effect of 1 and 2% silver are probably due to the silver part of the nanofiber-silver complex. It was reported that colloidal silver caused significant changes in physiological and biochemical processes in *Lemna gibba* L. [52]. The authors observed a concentration- and time-dependent oxidative stress, resulting in elevated total phenol content and activity of the antioxidant enzymes (SOD, CAT, APX, and GPO). Similarly, Sharma et al. [53] established that AgNPs improved the growth of Brassica juncea by controlling their antioxidant defense system.

Hydrogen peroxide content was strongly reduced in plants grown on a medium supplemented with NF-1%Ag and NF-2%Ag compared to control and with 0.5 mg L^−1^ BAP. This corresponds with the higher activities of the enzymes CAT, APX, and GPX, which neutralize H_2_O_2_, and lower content of the stress markers content (MDA, H_2_O_2,_ and proline). The only exception was the treatment with the highest concentration (100 mg L^−1^) of NF-2%Ag, where a significant increase in H_2_O_2_ content was observed. It may be suggested that the antioxidant system cannot compensate for the production of ROS induced by 100 mg L^−1^ NF-2%. El-Mahdy et al. [54] established that both AgNPs and AgNO_3_ at low concentrations improved the growth and development of banana plants *in vitro*, whereas high doses (100 and 200 mg L^−1^) possessed a phytotoxic effect. The authors reported a remarkable increase in H_2_O_2_ content and the activity of SOD, CAT, and GPO, as well as suppressed plant growth at these concentrations of AgNPs and AgNO_3_.

Proline and free-thiol-groups-containing compounds are an important part of the plant defense system. NF enriched with 1% colloidal Ag caused drastic elevation in proline content, whereas treatment with NF-2%Ag led to a less increase in the amount of low-molecular thiols. Shaikhaldein et al. [55] reported that AgNPs at concentrations of 40 and 50 mg L^−1^ increased proline content, and the level of the activities of SOD and CAT in Maerua oblongifolia raised *in vitro*. Thiols play an important role in heavy metal sequestration [56]. A significant increase of total thiols content in *in vitro* cultivated Zea mays in response to copper stress was reported [57].

Some amino acids, such as glutamic acid and proline, enhance SGs production [58,59]. A positive effect of casein hydrolysate, a complex organic extract comprising up to 18 amino acids, on stevioside and rebaudioside A accumulation has also been reported [60].

The questions and answers raised by this study will be a fundamental basis for further research on implementing technologies with a positive effect on pharmaceuticals and modern health management technologies. This knowledge will be used by researchers and related industries in this field to further study the biological activities, such as antibacterial, antitumor, and antioxidant, of phytochemicals.

## 4. Material and Methods

### 4.1. Chemical Synthesis

Although synthesis of the target organic compound has been described previously [26], here we use another route to obtain it (Figure 1). Its synthesis is presented below. Two organic compounds have been isolated: a targeted and an intermediate one. Chemicals used for this purpose were purchased either from Sigma-Aldrich, USA, Missouri, Jefferson: Nicotinic acid, Trifluoroacetic acid (TFA), N-diisopropylethylamine (DIPEA) and 1,6-diaminohexane, or Iris Biotech, Germany, Marktredwitz: Boc-L-Val-OH, dimethylformamide (DMF), 2-(1H-Benzotriazole-1-yl)-1,1,3,3-tetramethylaminium tetrafluoroborate (TBTU). Chemical synthesis was done in two consequent stages. First one: Synthesis of the precursor-N,N’-Bis-(Boc-L-Valyl)-1,6-diaminohexane (referred to as Boc-Val6).

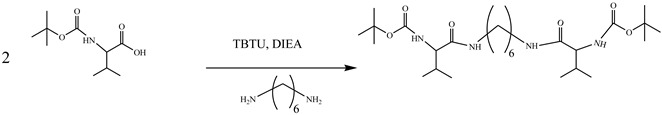



Hexamethylenediamine (1 eq) and Boc-L-Val-OH (2 eq) were dissolved together in a minimum amount of DMF at room temperature, then TBTU (2 eq) was added, followed by dropping DIPEA (2 eq). Obtained in this way reaction mixture was stirred overnight at room temperature. Afterward, 1 M NaHCO_3_ was added until a white precipitate formed, and the mixture was left in the refrigerator for 1–2 h so that the precipitate consolidated. Then the product was filtered off and washed firstly with 1 M NaHCO_3_, followed by washing with 10% citric acid and finally with cool distilled water until neutral pH of the washing water. The product was obtained after filtration as white crystals. The solid crude was recrystallized from ethylacetate.

Second step: the synthesis of M6 was done in two steps by deprotection of Boc-Val6 and in situ condensation with nicotinic acid.

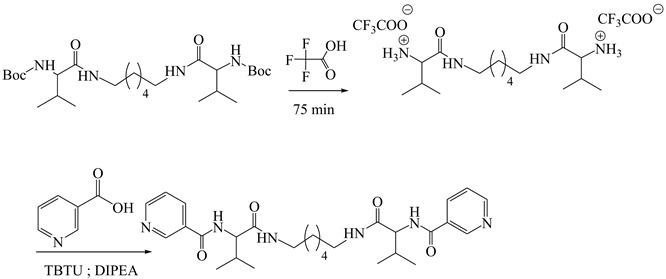



The mixture of Boc-Val6 (1 eq) and trifluoroacetic acid (TFA) (24 eq) was stirred for about 75 min. The excess TFA was removed by vacuum evaporation. The solid obtained was dissolved in min DMF (1g solid in 5–10 mL DMF) and DIEA was dropped in it so that pH 7 was achieved. To this solution, nicotinic acid (2.4 eq), TBTU (2.4 eq), and DIEA (2.4 eq) were added and dissolved, successively adjusting the amount of DMF as solvent. The reaction was left stirring overnight at room temperature. Then 1 M NaHCO_3_ was added to the reaction mixture so that the product as white precipitate was observed. The flask was left at room temperature for 1 h and then moved in the refrigerator at 4–6 °C for 1–2 h where the precipitate consolidates. The product was filtered off and washed with 1 M NaHCO_3_, then with cool distilled water until neutral pH of the washing water.

### 4.2. Analyses of the Newly Obtained Compound Were Done by NMR

^1^H and ^13^C spectra were recorded on Bruker Avance AV-II+-600 MHz spectrometer. The ^1^H and ^13^C NMR chemical shifts are given relative to TMS. Chemical shifts are expressed in ppm and coupling constants in Hz.

### 4.3. Nanofibers Synthesis and Enrichment with Colloidal Silver

For the purpose of colloidal silver loading, a product produced by Argenol Laboratorios, Spain, has been used. The stock solution of 20 mg mL^−1^ in distilled water was prepared before use. Compound M6 was dissolved in ethanol then an adequate amount of freshly prepared colloidal-silver stock solution was added. Then the mixture was left at the rotary evaporator for solvent removal, and the solid obtained was used for plant treatment. 1% (*w*/*w*) and 2% (*w*/*w*) colloidal silver were loaded into the organic compound.

UV-VIS studies of diluted aqua solution of AgNPs and ethanol solution of organic material with 2% Ag nanoparticles were performed using a two-channel spectrophotometer, model “Cary-100” of the company “VÁRIAN”, in the range (190–900) nm.

The prepared organic nanofibers with incorporated 2% Ag nanoparticles have been investigated by a Fourier Transform Spectrophotometer EQUINOX 55 (Brucker, Billerica, MA, USA) in the mid-IR region (4000–400 cm ^−1^) applying a standard technique with KBr pellets.

The morphology of the as-prepared samples was inspected with a scanning electron microscope (LYRA I XMU, Tescan [Brno, Czech Republic]).

### 4.4. Plant Material

Seeds from *S. rebaudiana* Bert were purchased from the commercial seed source Company “Stevia-Paraguay“. The seeds were surface sterilized by soaking in 70% ethanol for 2 min and then treated with 15% bleach solution (commercial bleach containing 4.85% sodium hypochlorite) for 15 min and again washed three times each for 15 min in sterilized distilled water to remove the traces of commercial bleach. For *in vitro* seed germination, *S. rebaudiana* seeds were cultured on an MS medium including vitamins supplemented with 3.0% sucrose, 7.0 g L^−1^ agar and 0.4 mg L^−1^ gibberellic acid, and 1.0 mg L^−1^ CaCl_2_ for three weeks of culture. The *in vitro* culture conditions were maintained according to Zayova et al. [61]. For shoot induction and proliferation nodal explants were used. Stem explants (1 cm) were excised from three months *in vitro* shoots and placed onto MS medium supplemented with various concentrations (1, 10, 50, 100 mg L^−1^) of aminoacid nanofibers enriched with 1% and 2% colloidal Ag particles (NF-1%Ag, NF-2%Ag). The control plantlets were developed on two nutrient media—MS free of PGR and other additives and MS medium containing 0.5 mg L^−1^ BAP (C + BAP), selected as highly effective for shoot proliferation in our previous study [62]. Twenty stem explants were placed on each of the ten medium variants, and each treatment was repeated twice. The mean number of shoots per explant, shoot length, root length, and fresh weight of shoots and roots were assessed after 4 weeks of culture. Cultures were incubated at 25 ± 2 °C under cool-white fluorescent light (Philips) with a 16 h photoperiod at an intensity of 40 μmol m^−2^ s^−1^.

### 4.5. Antioxidant Capacity

The enzymes with antioxidant potential superoxide dismutase (SOD), catalase (CAT), ascorbate peroxidase (APX), and guaiacol peroxidase (GPO) were extracted from the plant samples following the method of Hristozkova et al. [63]. Total SOD (EC 1.15.1.1), activity [64], CAT (EC 1.11.1.6) activity [65], APX (EC 1.11.1.1) activity [66], and GPO (EC 1.11.1.7) activity [67] were determined spectrophotometrically on UV/VIS spectrophotometer (Shimadzu UV-1601, Japan, Tokyo). Soluble protein content was determined by using bovine serum albumin as a standard [68].

To analyze the antioxidant compounds, 0.3 g dry samples from four weeks *in vitro* grown plantlets were ground and suspended in 80% (*v/v*) aqueous methanol. The resulting filtrates were pooled for further processing. Concentrations of total phenolic compounds were determined spectrophotometrically using the Folin–Ciocalteu reagent and calculated as caffeic acid equivalents [69]. Total flavonoid content in plant tissues was measured spectrophotometrically using the standard curve of catechin [70]. The percentage of radical scavenging activity was measured by color artificial stable free radical DPPH• (1,1-diphenyl-2-picrylhydrazyl) assay. The changes in color (from deep violet to light yellow) were read at a 517 nm UV/VIS-spectrophotometer (Shimadzu, Japan, Tokyo) [71]. The ferric reducing antioxidant power (FRAP method) depends upon the level of reduction of the ferric tripyridyltriazine (Fe(III)-TPTZ) complex to the ferrous tripyridyltriazine (Fe(II)-TPTZ) by a reductant at low pH [72]. Spectrophotometric quantification was used to determine water-soluble (WS-AOM) and lipid-soluble (LS-AOM) metabolites with antioxidant capacity, expressed as equivalents of ascorbate and α-tocopherol [73]. This method is based on reducing Mo (VI) to Mo (V) by the sample analysis and the subsequent formation of a blue-green phosphomolybdenum complex at acidic pH.

### 4.6. Stress Markers Content Analyses

Fresh leaf material (300 mg) was homogenized with 0.1% (*w*/*v*) trichloroacetic acid for the determination of proline, hydrogen peroxide (H_2_O_2_), malondialdehyde (MDA), and free-thiol-groups-containing compounds (SH-) content. Free proline was derivatized with acid ninhydrin, and absorbance was read at 520 nm, according to Bates et al. [74]. Malondialdehyde content was determined as a thiobarbituric acid-reagent product, according to Kramer et al. [75], by using the extinction coefficient of 155 mM^−1^ cm^−1^. Hydrogen peroxide content was estimated spectrophotometrically [76]. The content of free-thiol-groups-containing compounds was determined by incubating 40 μL supernatant with 150 μL Ellman’s reagent for 10 min at room temperature [77]. The absorbance was read at 412 nm.

### 4.7. Stevioside, Rebaudioside A, and Soluble Sugar Analyses

For sample preparation for HPLC analysis, 50 mg of dried and powdered leaves were extracted with 5 mL of water at 40 °C in an ultrasonic bath for 30 min. The obtained extracts were centrifuged, filtered, and transferred to a volumetric flask, and methanol was added up to 5 mL. The extracts were treated with solid phase extraction (SPE) cartridges filled with C18 sorbents according to the procedure described by Bergs et al. [78].

The HPLC analysis was performed on Shimadzu Nexera-i LC-2040C 3D Plus liquid chromatograph equipped with a photodiode array detector (Shimadzu, Japan, Tokyo), analytical column Intersil NH_2_ (3 μm × 4.0 × 150 mm) (GL Sciences, Japan, Tokyo), wavelength 210 nm, mobile phase CH_3_CN:H_2_O in gradient mode, the oven temperature at 40 °C, a flow rate of 0.8 mL min^−1^ and injection volume of 4 µL.

Stevioside and rebaudioside A (Phytolab GmbH & Co. KG, Vestenbergsgreuth, Germany) were used as external standards. The quantification was performed using analytical standard curves prepared by mixing authentic standards at concentrations from 0.075 to 1.0 mg mL^−1^.

All solvents were of analytical grade. Ascorbate, guaiacol, hydrogen peroxide, DPPH, and ammonium molybdate were obtained from Merck (Darmstadt, Germany). Nitroblue tetrazolium, riboflavin, and methionine were purchased from Sigma (Jefferson, MO, USA). All other chemicals were of analytical grade.

Reducing sugars were analyzed by the phenol-sulphuric acid procedure by Ashwell [79].

### 4.8. Statistical Analysis

The data were statistically processed by analysis of variance (one-way ANOVA analyses) for comparison of means, and significant differences were calculated according to Fisher’s least significance difference (LSD) test at the 5% significance level using a statistical software package (Statgraphics Plus, version 5.1 for Windows, (1994) Statistical Graphics Corporation, Warrenton, VA, USA).

## 5. Conclusions

The results obtained from these analyzes make it possible to conclude that nanofibers from a low molecular compound derivative of amino acid Valine as promising carriers of 1% and 2% colloidal silver particles added to the MS media possessed a hormetic effect. At low concentrations, from 1 to 50 mg L^−1^, they have a beneficial impact on plant growth, but at high concentrations, 100 mg L^−1,^ they have a harmful effect. The highest amount of stevioside was achieved in plants treated with 100 mg L^−1^ NF-1% Ag.

This study provides the first evidence of the hormetic effect of the enriched with colloidal Ag particles nanofibers on the culture by direct organogenesis on the development and production of natural antioxidants in *S. rebaudiana*. The use of nanofibers, formed by peptidomimetics carriers of the biologically active agent silver particles, can help in the *in vitro* growth and production of stevia, offering the possibility for its introduction into agriculture.

## Figures and Tables

**Table 1:** Morphological parameters of *in vitro* grown *Stevia rebaudiana* plantlets on PGR-free medium and medium supplemented with BAP and amino acid nanofibres in various concentrations (1, 10, 50, 100 mg L^−1^) enriched with 1% and 2% colloidal Ag (NF-1%Ag, NF-2%Ag).

Treatments	Shoots FW	Shoot Length	Shoots Number	Rooting	Arcsine Transformation
	g Plant^−1^	cm	Explant^−1^	%	for Rooting %
Control	0.115 ± 0.005a	5.97 ± 0.29ab	1.01 ± 0.05a	0.04	0.031
C + BAP	0.353 ± 0.017de	7.58 ± 0.37e	1.70 ± 0.08d	0.00	0.000
	
1 mg L^−1^ NF-1% Ag	0.305 ± 0.015c	6.83 ± 0.34cd	1.39 ± 0.06bc	15.81	0.652
10 mg L^−1^ NF-1% Ag	0.334 ± 0.017d	7.30 ± 0.36de	1.34 ± 0.06bc	18.30	0.712
50 mg L^−1^ NF-1% Ag	0.371 ± 0.019e	8.39 ± 0.42f	1.45 ± 0.07bc	42.92	1.571
100 mg L^−1^ NF-1% Ag	0.285 ± 0.014c	6.58 ± 0.32bc	1.40 ± 0.07c	37.12	1.194
	
1 mg L^−1^ NF-2% Ag	0.377 ± 0.019e	8.28 ± 0.41f	2.44 ± 0.12e	13.68	0.600
10 mg L^−1^ NF-2% Ag	0.412 ± 0.021f	8.63 ± 0.17f	3.35 ± 0.16f	17.13	0.684
50 mg L^−1^ NF-2% Ag	0.464 ± 0.023g	10.79 ± 0.53g	3.25 ± 0.16f	28.43	0.951
100 mg L^−1^ NF-2% Ag	0.193 ± 0.010b	5.35 ± 0.26a	1.25 ± 0.06a	26.00	0.892
LSD	0.029	0.657	0.172		

Values are means ± SE, n = 20; different letters indicate significant differences assessed by the Fisher LSD test (*p* ≤ 0.05) after performing ANOVA one-way analysis. We use the letter ‘a’ for the lowest data value and ascend to the next letters for higher data value.

**Table 2:** Stevioside, rebaudioside A, and sugars contained in the *Stevia rebaudiana* plantlets *in vitro* propagated on MS medium free of BAP, on MS medium with BAP, and MS medium supplemented with various concentrations (1, 10, 50, 100 mg L^−1^) of amino acid nanofibers enriched with 1% and 2% colloidal Ag (NF-1%Ag, NF-2%Ag).

Treatments	Stevioside	Rebaudioside A	Sugars
	mg g DW^−1^	mg g DW^−1^	mg g FW^−1^
C	18.611 ± 0.069 ^h^	10.474 ± 0.069 ^g^	15.25 ± 0.76 ^a^
C + BAP	12.401 ± 0.093 ^c^	4.018 ± 0.023 ^a^	16.02 ± 0.75 ^a^
			
1 mg L^−1^ NF-1% Ag	18.297 ± 0.023 ^g^	6.802 ± 0.023 ^c^	21.89 ± 0.84 ^b^
10 mg L^−1^ NF-1% Ag	16.515 ± 0.119 ^d^	11.600 ± 0.024 ^h^	18.28 ± 0.80 ^a^
50 mg L^−1^ NF-1% Ag	18.021 ± 0.046 ^f^	7.015 ± 0.023 ^cd^	16.07 ± 0.71 ^a^
100 mg L^−1^ NF-1% Ag	23.464 ± 0.023 ^i^	6.133 ± 0.023 ^b^	18.68 ± 0.76 ^ab^
			
1 mg L^−1^ NF-2% Ag	9.048 ± 0.023 ^a^	3.580 ± 0.046 ^a^	28.54 ± 1.12 ^c^
10 mg L^−1^ NF-2% Ag	12.316 ± 0.225 ^c^	7.537 ± 0.017 ^de^	49.00 ± 2.36 ^d^
50 mg L^−1^ NF-2% Ag	11.197 ± 0.046 ^b^	7.858 ± 0.023 ^e^	53.27 ± 2.12 ^e^
100 mg L^−1^ NF-2% Ag	17.337 ± 0.047 ^c^	9.235 ± 0.047 ^f^	94.92 ± 4.27 ^f^
LSD	0.158	0.060	3.508

Values are means ± SE, n = 6; different letters indicate significant differences assessed by the Fisher LSD test (*p* ≤ 0.05) after performing ANOVA one-way analysis. We used the letter ‘a’ for the lowest data value and ascending to the next letters for higher-data value.

## Data Availability

Not applicable.

## Supplementary Materials

The following supporting information can be downloaded at: https://www.mdpi.com/article/10.3390/plants11192468/s1. Figure S1: *S. rebaudiana* plantlets *in vitro* propagated on MS medium (A control plants); MS medium containing 0.5 mg L^−1^ BAP (B); MS medium with various concentrations (1, 10, 50, 100 mg L^−1^) of aminoacid nanofibers enriched with 1% (C, D, E, F) and 2% colloidal (G, H, I, J) Ag particles (NF-1%Ag, NF-2%Ag).

## References

[1] International Plant Names Index The Royal Botanic Gardens, Kew, Harvard University Herbaria & Libraries and Australian National Botanic Gardens. https://powo.science.kew.org/.

[2] Geuns J.M.C. (2003). Stevioside. Phytochemistry.

[3] Singh S.D., Rao G.P. (2005). Stevia: The herbal sugar of 21st century. Sugar Tech..

[4] Felippe G.M., Lucas N.M.C. (1971). Estudo da viabilidade dos frutos de *Stevia rebaudiana* Bert. Hoehnea..

[5] Carneiro J.W.P., Muniz A.S., Guedes T.A. (1997). Greenhouse bedding plant production of* Stevia rebaudiana* (Bert) Bertoni. Can. J. Plant Sci..

[6] Kaplan B., Duraklioglu S., Turgut K. (2019). Sustainable micropropagation of selected *Stevia rebaudiana* Bertoni genotypes. Acta Sci. Polon.-Hort. Cult..

[7] Yesmin S. (2019). In vitro micropropagation of *Stevia rebaudiana* Bertoni. Plant Tiss. Cult Biotechnol..

[8] Thiyagarajan M., Venkatachalam P. (2012). Large scale *in vitro* propagation of *Stevia rebaudiana* (Bert) for commercial application: Pharmaceutically important and anti-diabetic medicinal herb. Ind. Crops Prod..

[9] Uddin M.S., Chowdhury M.S.H., Khan M.M.M.H., Ahmed R., Baten M.A. (2006). In vitro propagation of *Stevia rebaudiana*, Bert. in Bangladesh. Afr. J. Biotechnol..

[10] Rafiq M., Dahot M.U., Mangrio S.M., Naqri H.A., Qarshi I.A. (2007). In vitro clonal propagation and biochemical analysis of field established *Stevia rebaudiana* Bertoni. Pak. J. Bot..

[11] Sairkar P., Chandravanshi M.K., Shukla N.K., Mehrotra M.N. (2009). Mass propagation of an economically important medicinal plant *Stevia rebaudiana* using in vitro propagation technique. J. Med. Plants Res..

[12] Ozyigit I.I. (2008). Phenolic changes during in vitro organogenesis of cotton (*Gossypium hirsutum* L.) shoot tips. Afr. J. Biotechnol..

[13] Reis E., Batista M.T., Canhoto J.M. (2008). Effect and analysis of phenolic compounds during somatic embryogenesis induction in *Feijoa sellowiana* Berg. Protoplasma.

[14] Abdi G., Salehi H., Khosh-Khui M. (2008). Nano_Silver: A novel nanomaterial for removal of bacterial contaminants in valerian (*Valeriana officinalis* L.) tissue culture. Acta Physiol. Plant..

[15] Giridhar P., Reddy B., Ravishankar G.A. (2001). Silver nitrate influences in vitro shoot multiplication and root formation in *Vanilla planifolia* Andr. Curr. Sci..

[16] Cristea T.O., Leonte C., Brezeneau C., Brezeanu M., Ambarus S., Calinand M., Prisecaru M. (2012). Effect of AgNO_3_ on androgenesis of *Brassica oleraceae* L. anthers cultivated in vitro. Afr. J. Biotechnol..

[17] Tamimi S.M. (2015). Effects of ethylene inhibitors, silver nitrate (AgNO3), cobalt chloride (CoCl2) and aminooxyacetic acid (AOA), on in vitro shoot induction and rooting of banana (*Musa acuminata* L.). Afr. J. Biotechnol..

[18] Bais H.P., Ravishankar G. (2002). Role of polyamines in the ontogeny of plants and their biotechnological applications. Plant Cell Tissue Organ Cult..

[19] Jakubowicz M., Gałgańska H., Nowak W., Sadowski J. (2010). Exogenously induced expression of ethylene biosynthesis, ethylene perception, phospholipase D, and Rboh-oxidase genes in broccoli seedlings. J. Exp. Bot..

[20] Arraes F.B.M., Beneventi M.A., de Sa M.E.L., Paixao J.F.R., Albuquerque E.V.S., Marin S.R.R., Purgatto E., Nepomuceno A., Grossi-de-Sa M. (2015). Implications of ethylene biosynthesis and signalling in soybean drought stress tolerance. BMC Plant Biol..

[21] Nissen P. (1994). Stimulation of somatic embryogenesis in carrot by ethylene: Effects of modulators of ethylene biosynthesis and action. Physiol. Plant..

[22] Kumar S.V., Rajam M.V. (2004). Polyamine ethylene nexus: A potential target for post harvest biotechnology. Ind. J. Biotechnol..

[23] Golkar P., Moradi M., Garousi G.A. (2019). Elicitation of stevia glycosides using salicylic acid and silver nanoparticles under callus culture. Sugar Tech..

[24] Tarrahi R., Mahjouri S., Khataee A. (2021). A review on in vivo and in vitro nanotoxicological studies in plants: A headlight for future targets. Ecotoxicol. Environ. Saf..

[25] Castro-González C., Sánchez-Segura L., Gómez-Merino F., Bello-Bello J. (2019). Exposure of stevia (*Stevia rebaudiana* B.) to silver nanoparticles in vitro: Transport and accumulation. Sci. Rep..

[26] Akhtar G., Jaskani M.J., Sajjad Y., Akram A. (2016). Effect of antioxidants, amino acids and plant growth regulators on in vitro propagation of *Rosa centifolia*. Iran. J. Biotechnol..

[27] Tsekova D.S., Escuder B., Miravet J.F. (2008). Solid-state polymorphic transition and solvent-free self-assembly in the growth of organic crystalline microfibers. Cryst. Growth Des..

[28] Miravet J.F., Escuder B. (2005). Pyridine-functionalised ambidextrous gelators: Towards catalytic gels. Chem. Commun..

[29] Roy G., Miravet J.F., Escuder B., Sanchez C., Llusar M.J. (2006). Morphology templating of nanofibrous silica through pH-sensitive gels: “in situ” and “post-diffusion” strategies. J. Mater. Chem..

[30] Tantcheva V.V., Petkov G., Karamukova S., Abarova Y., Chekalarova D., Tsekova B., Escuder J., Miravet K., Lyubomirova N. (2006). Neuropharmacological activity of newly synthesized derivatives of valin L.. Bulg. Chem. Commun..

[31] Vishwakarma K., Shweta, Upadhyay N., Singh J., Liu S., Singh V.P., Prasad S.M., Chauhan D.K., Tripathi D.K., Sharma S. (2017). Differential phytotoxic impact of plant mediated silver nanoparticles (AgNPs) and silver nitrate (AgNO3) on *Brassica* sp.. Front. Plant Sci..

[32] Chi G.L., Pua E.C. (1989). Ethylene inhibitors enhanced de novo shoot regeneration from cotyledons of *Brassica campastris* spp. in vitro. Plant Sci..

[33] Petrova M., Zayova E., Vitkova A. (2011). Effect of silver nitrate on in vitro root formation of *Gentiana lutea*. Rom. Biotechnol. Lett..

[34] Lei C., Wang H., Liu B., Ye H. (2014). Effects of silver nitrate on shoot regeneration of *Artemisia annua* L.. Plant Biotechnol..

[35] Kumar V., Parvatam G., Ravishankar G.A. (2009). AgNO_3_-a potential regulator of ethylene activity and plant growth modulator. Electron. J. Biotechnol..

[36] Zhao X.C., Qu X., Mathews D.E., Schaller G.E. (2002). Effect of ethylene-pathway mutations upon expression of the ethylene receptor ETR1 from Arabidopsis. Plant Physiol..

[37] Almutairi Z.M., Alharbi A. (2015). Effect of silver nanoparticles on seed germination of crop plants. J. Adv. Agric..

[38] Pallavi, Mehta C.M., Srivastava R., Arora S., Sharma A.K. (2016). Impact assessment of silver nanoparticles on plant growth and soil bacterial diversity. 3 Biotech.

[39] Agathokleous E., Kitao M., Calabrese E.J. (2019). Hormesis: A compelling platform for sophisticated plant science. Trends Plant Sci..

[40] Islam S., Rahman I.A., Islam T., Ghosh A. (2017). Genome-wide identification and expression analysis of glutathione S-transferase gene family in tomato: Gaining an insight to their physiological and stress-specific roles. PLoS ONE.

[41] Peres N.A., Oliveira M.S., MacKenzie S.J. Colletotrichum Crown rot (*Anthracnose crown* rot) of Strawberries. *UF/IFAS* Extension 2017, 238–240. http://edis.ifas.ufl.edu.

[42] Röck-Okuyucu B., Bayraktar M., Akgun I.H., Gurel A. (2016). Plant growth regulator effect ts on in vitro propagation and stevioside production in *Stevia rebaudiana* Bertoni. HortScience.

[43] Brandle J.E., Starratt A.N., Gijzen M. (1998). *Stevia rebaudiana*: Its agricultural, biological, and chemical properties. Can. J. Plant Sci..

[44] Brandle J.E., Telmer P.G. (2007). Steviol glycoside biosynthesis. Photochemistry.

[45] Ramezani M., Asghari S., Gerami M., Ramezani F., Abdolmaleki M.K. (2019). Effect of silver nanoparticle treatment on the expression of key genes involved in glycosides biosynthetic pathway in *S. rebaudiana* B. Plant. Sugar Tech..

[46] Nokandeh S., Ramezani M., Gerami M. (2021). The physiological and biochemical responses to engineered green graphene/metal nanocomposites in *Stevia rebaudiana*. J. Plant Biochem. Biotechnol..

[47] Ahmad P., Latef A., Hashem A., Abd_Allah E.F., Gucel S., Tran L. (2016). Nitric oxide mitigates salt stress by regulating levels of osmolytes and antioxidant enzymes in chickpea. Front. Plant Sci..

[48] Rosa M., Prado C., Podazza G., Interdonato R., González J.A., Hilal M., Prado F.E. (2009). Review Soluble sugars—Metabolism, sensing and abiotic stress A complex network in the life of plants. Plant Signal. Behav..

[49] Agnez-Lima L.F., Melo J.T.A., Silva A.E., Oliveira A., Timoteo A., Lima-Bessa K., Martinez G.R., Medeiros M., Di Mascio P., Galhardo R. (2012). DNA damage by singlet oxygen and cellular protective mechanisms. Mutat. Res. Rev. Mutat. Res..

[50] Smulders M., De Klerk G. (2011). Epigenetics in plant tissue culture. Plant Growth Regul..

[51] Fazal H., Abbasi B.H., Ahmad N., Ali S., Akbar F., Kanwal F. (2016). Correlation of different spectral lights with biomass accumulation and production of antioxidant secondary metabolites in callus cultures of medicinally important *Prunella vulgaris* L.. J. Photochem. Photobiol. B Biol..

[52] Varga M., Horvatić J., Barišić L., Lončarić Z., Sikirić M., Erceg I., Kočić A., Čamagajevac I. (2019). Physiological and biochemical effect of silver on the aquatic plant *Lemna gibba* L.: Evaluation of commercially available product containing colloidal silver. Aquat. Toxicol..

[53] Sharma P., Bhatt D., Zaidi M.G.H., Saradhi P.P., Khanna P.K., Arora S. (2012). Silver nanoparticle-mediated enhancement in growth and antioxidant status of *Brassica juncea*. Appl. Biochem. Biotechnol..

[54] El-Mahdy M.T.K., Radi A., Shaaban M. (2019). Impacts of exposure of banana to silver nanoparticles and silver ions in vitro. Middle East J. Appl. Sci..

[55] Shaikhaldein H., Al-Qurainy F., Nadeem M., Khan S., Tarroum M., Salih A.M. (2020). Biosynthesis and characterization of silver nanoparticles using *Ochradenus arabicus* and their physiological effect on *Maerua oblongifolia* raised in vitro. Sci. Rep..

[56] Ederli L., Reale L., Ferranti F., Pasqualini S. (2004). Responses induced by high concentration of cadmium in *Phragmites australis* roots. Physiol. Plant..

[57] Aly A.A., Mohamed A.A. (2012). The impact of copper ion on growth, thiol compounds and lipid peroxidation in two maize cultivars (*Zea mays* L.) grown in vitro. Aust. J. Crop Sci..

[58] Hendawey M.H., Abo El Fadl R.E. (2014). Biochemical studies on the production of active constituents in *Stevia rebaudiana* L.. callus. Glob. J. Biotechnol. Biochem..

[59] Esmaeili H., Karami A., Maggi F. (2018). Essential oil composition, total phenolic and flavonoids contents, and antioxidant activity of *Oliveria decumbens* Vent. (Apiaceae) at different phenological stages. J. Clean. Prod..

[60] Bayraktar M., Okuyucu B., Akgun I., Fedekar S., Kanik H., Gurel A. (2015). Determination of clone lines having a high content of stevioside and rebaudioside a for in vitro commercial production of *Stevia rebaudiana*. J. AARI.

[61] Zayova E., Geneva M., Dimitrova L., Miladinova-Georgieva K., Hristozkova M., Stancheva I. (2019). Impact of plant growth regulators on Greek oregano micropropagation and antioxidant activity. Biosci. Biotechnol. Res. Asia.

[62] Zayova E., Petrova M., Vasilevska-İvanova R., Stoeva D., Krapchev B. (2013). A tissue culture technique for propagation of *Paulownia elongata* tree. Biyolojik Çeşitlilik ve Koruma.

[63] Hristozkova M., Geneva M., Stancheva I., Iliev I., Azcón-Aguilar C. (2017). Symbiotic association between golden berry (*Physalis peruviana*) and arbuscular mycorrhizal fungi in heavy metal-contaminated soil. J. Plant Prot. Res..

[64] Giannopolitis C.N., Reis S.K. (1997). Superoxide dismutase: I. Occurrence in higher plants. Plant Physiol..

[65] Upadhyaya A., Sankhla D., Davis T., Sankhla N., Smith B. (1985). Effect of paclobutrazol on the activities of some enzymes of activated oxygen metabolism and lipid peroxidation in senescing soybean leaves. J. Plant Physiol..

[66] Nakano Y., Asada K. (1987). Purification of ascorbate peroxidase in spinach chloroplasts: Its inactivation in ascorbate-depleted medium and reactivation by monodehydroascorbate radical. Plant Cell Physiol..

[67] Lagrimini M., Gingas V., Finger F., Rothstein S., Liu T.-T.Y. (1997). Characterization of antisense transformed plants deficient in the tobacco anionic peroxidase. Plant Physiol..

[68] Bradford M.M. (1976). A rapid and sensitive method for the estimation of microgram quantities of protein utilizing the principle of protein-dye binding. Anal. Biochem..

[69] Pfeffer H., Dannel F., Römheld V. (1998). Are there connection between phenol metabolism, ascorbate metabolism and membrane integrity in leaves of boron-deficient sunflower plants?. Physiol. Plant..

[70] Zhishen J., Mengcheng T., Jianming W. (1999). The determination of flavonoid contents in mulberry and their scavenging effects on superoxide radicals. Food Chem..

[71] Tepe B., Sokmen M., Akpulat H.A., Sokmen A. (2006). Screening of the antioxidant potentials of six Salvia species from Turkey. Food Chem..

[72] Benzie I., Strain J. (1996). The ferric reducing ability of plasma (FRAP) as a measure of “antioxidant power”: The FRAP assay. Anal. Biochem..

[73] Prieto P., Pineda M., Aguilar M. (1999). Spectrophotometric quantitation of antioxidant capacity through the formation of a phosphomolybdenum complex: Specific application to the determination of vitamin E. Anal. Biochem..

[74] Bates L., Waldren R., Teare I.D. (1973). Rapid determination of free proline for water-stress studies. Plant Soil.

[75] Kramer G.F., Norman H., Krizek D., Mirecki R. (1991). Influence of UV-B radiation on polyamines, lipid peroxidation and membrane lipids in cucumber. Phytochemistry.

[76] Alexieva V., Sergiev I., Mapelli S., Karanov E. (2001). The effect of drought and ultraviolet radiation on growth and stress markers in pea and wheat. Plant Cell Environ..

[77] Ellman G.l. (1959). Tissue sulphydryl groups. Arch. Biochem. Biophys..

[78] Bergs D., Burghoff B., Joehnck M., Martin G., Schembecker G. (2012). Fast and isocratic HPLC-method for steviol glycosides analysis from *Stevia rebaudiana* leaves. J. Verbrauch. Lebensm..

[79] Ashwell G., Neufeld E.F., Ginsburg V. (1966). New colorimetric methods of sugar analysis. Methods in Enzymology.

