# Supplementary material for: Improvement of Stevia rebaudiana Bertoni In Vitro Propagation and Steviol Glycoside Content Using Aminoacid Silver Nanofibers
Source: Plants (Basel). 2022 Sep 21;11(19):2468. doi: 10.3390/plants11192468 (PMC9572510; doi:10.3390/plants11192468)
Supplement: Supplementary file 1 [file plants-11-02468-s001.zip › Figure S1.pdf]

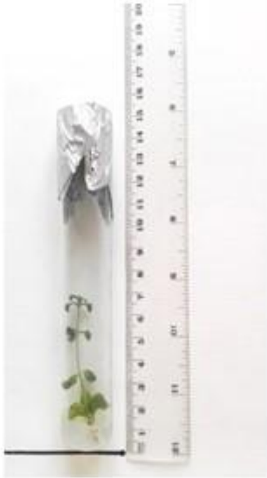

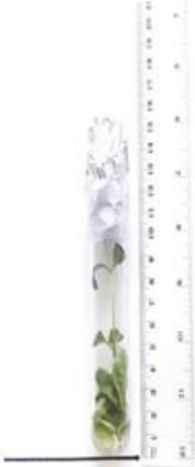

A B

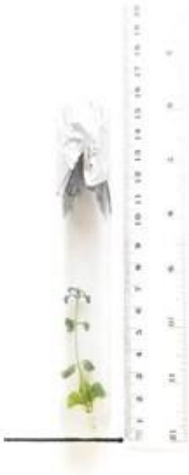

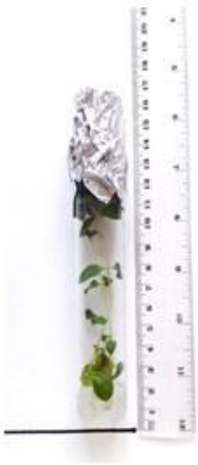

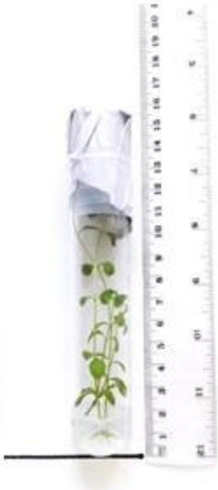

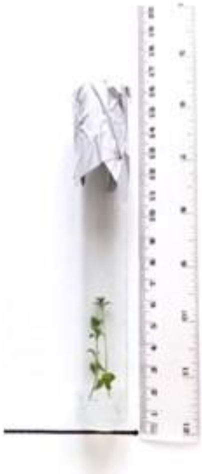

C D E F

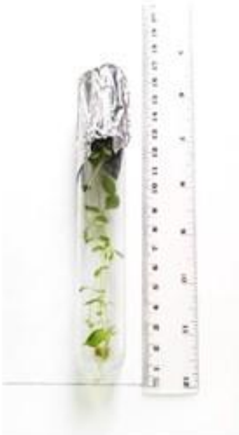

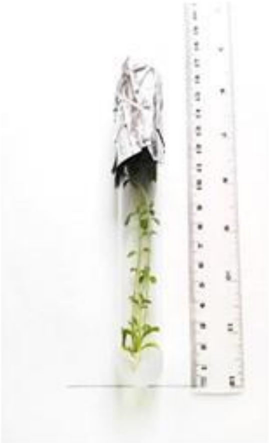

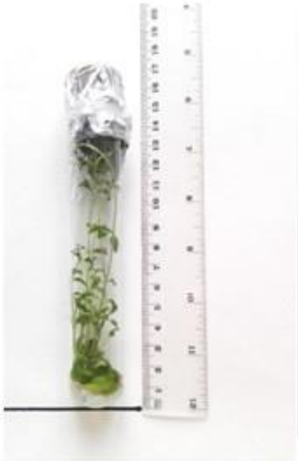

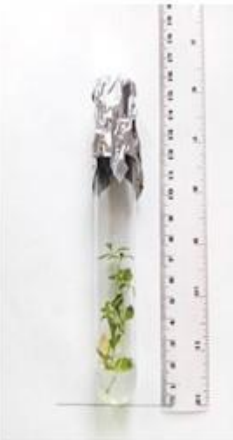

G H I J

Figure S1. *S. rebaudiana* plantlets *in vitro* propagated on MS medium (**A** control plants); MS medium containing 0.5 mg L^{-1} BAP (**B**); MS medium with various concentrations (1, 10, 50, 100 mg L^{-1}) of aminoacid nanofibers enriched with 1% (**C**, **D**, **E**, **F**) and 2% colloidal (**G**, **H**, **I**, **J**) Ag particles (NF-1%Ag, NF-2%Ag).
